# Dr. John Moroney

**Published:** 1918-08

**Authors:** 


**Dr. John Moroney** of Auburn, died May 23, age 66. He graduated from the Long Island College Hospital in 1874.

